# Partnering with patients in healthcare research: a scoping review of ethical issues, challenges, and recommendations for practice

**DOI:** 10.1186/s12910-020-0460-0

**Published:** 2020-05-11

**Authors:** Joé T. Martineau, Asma Minyaoui, Antoine Boivin

**Affiliations:** 1 Department of Management, HEC Montreal, 3000 chemin de la Cote-Ste-Catherine, Montreal, QC H3T2A7 Canada; 2 University of Montreal, Montreal, Canada; 3 Canada Research Chair in Patient and Public Partnership, CHUM Research Center (CRCHUM) and University of Montreal, Montreal, Canada

**Keywords:** Scoping review, Ethical issues, Patient partnership, Patient engagement, Healthcare research

## Abstract

**Background:**

Partnering with patients in healthcare research now benefits from a strong rationale and is encouraged by funding agencies and research institutions. However, this new approach raises ethical issues for patients, researchers, research professionals and administrators. The main objective of this review is to map the literature related to the ethical issues associated with patient partnership in healthcare research, as well as the recommendations to address them. Our global aim is to help researchers, patients, research institutions and research ethics boards reflecting on and dealing with these issues.

**Methods:**

We conducted a scoping review of the ethical issues and recommendations associated with partnering with patients in healthcare research. After our search strategy, 31 peer reviewed articles published between 2007 and 2017 remained and were analyzed.

**Results:**

We have identified 58 first-order ethical issues and challenges associated with patient partnership in research, regrouped in 18 second-order ethical themes. Most of the issues are transversal to all phases and stages of the research process and a lot of them could also apply to patient-partnership in other spheres of health, such as governance, quality improvement, and education. We suggested that ethical issues and challenges of partnered research can be related to four ethical frameworks: 1) Research ethics; 2) Research integrity; 3) Organizational ethics, and 4) Relational ethics.

**Conclusions:**

We have identified numerous ethical issues associated with the recent approach of patient-partnership in research. These issues are more diverse than the issues associated with a more traditional research approach. Indeed, the current discussion on how we address ethical issues in research is anchored in the assumption that patients, as research participants, must be protected from risk. However, doing research with, and not on, the patient involves changes in the way we reflect on the ethical issues associated with this approach to research. We propose to broaden the ethical discussion on partnered research to not only rely on a research ethics framework, but to also frame it within the areas of research integrity, organizational ethics and relational ethics.

## Background

Patient engagement initiatives in different areas of healthcare have proliferated in the last decade [1–3]. Patients are now more commonly integrated in the governance of healthcare institutions, in quality improvement initiatives, education and policy making. In clinical settings, patients are sometimes integrated in multidisciplinary clinical teams, assisting professionals and supporting patients in their journey of care, in rehabilitation, and end-of-life care [3–5]. Lastly, patients are now more often engaged in healthcare research initiatives, not as research participants, but as research collaborators, partners or even as co-researchers [5, 6]. However, in these various initiatives, and despite all the good will of the various actors involved, there is still more than often a gap between the ideal conception of patient partnership in meaningful and fruitful initiatives and the actual practices of patient engagement in healthcare. Patient engagement efforts can sometimes be tainted by misconceptions, misunderstandings, and frustrations.

Carman et al. [1] describe patient engagement as “patients, families, their representatives, and health professionals working in active partnership at various levels across the health care system […] to improve health and health care” (p. 224). In the literature, patient engagement usually refers to working and engaging with patients, i.e. individuals with first-hand experience of health issue, or with their caregivers. Carman et al. [1] also describe different levels of patient participation, and propose a continuum of patient engagement in healthcare, ranging from patient consultation, to involvement, to partnership and shared leadership. In the research sphere, Marlett et al. [7] define patient engagement as “collaborative research that is done by, with and for patients to inform health care and health research decisions and questions” (p. 1058). Macaulay et al. [8] describe research partnership as “a mutually respectful relationship based on sharing responsibilities, costs, and benefits” (p. 775). Shippee et al. [6] distinguish between a passive engagement of patient in research, in which patients are research participants, and an active engagement, which corresponds to the patients-as-partner view proposed by Karazivan et al. [2]. In this latter view, patient engagement initiatives aim at engaging patients through an “equitable collaboration with individuals, families and communities affected by a health topic at all stages of the research process, from conception of the study idea through dissemination of results/findings" (p. 314) [9].

Engaging with patients in healthcare research now benefits from a strong ethical rationale, emphasizing that active participation of patients in research has the potential to empower patients, to ensure a more responsible research, and to add value [10], quality, effectiveness, and validity to the research process, among other potential benefits [11–13]. However, the ideal or normative conception of active patient engagement or partnership in research is often confronted, in practice, with a more pragmatic view. As mentioned before, full partnership is intended, in theory, as engagement of patients and co-leadership in all stages of research, but the extent to which this practice is really implemented in healthcare research projects can be challenged. Also, authors recognise that effective patient partnership in research requires additional efforts when compared with a more traditional research process [11]. Researchers often feel they are not well equipped and trained to face these issues [14]. Indeed, as interest, pressures and incentives to engage patients in research increase, researchers, patients, members of research ethics boards, research institutions, funding agencies, health organizations managers and professionals encounter more frequently questions and/or ethical dilemmas related to patient engagement in health research. Research with, and not on, the patient involves significant changes in the governance of research projects, in the composition of research teams, in the relationships between team members, as well as in the design, conduct and dissemination of research itself. In many cases, actors are confronted with the various issues that emerge from these changes, without any clear direction and support.

The main objective of this paper is thus to map the literature related to the ethical issues associated with this emergent practice of partnering with patients in healthcare research, and to identify the proposed recommendations, from the literature, to deal with these issues and challenges. We focus here on patient partnership and shared leadership in research, which corresponds to the highest level of patient engagement on Carman et al.’s [1] continuum of patient engagement in healthcare. A secondary objective is to identify the “gaps” in existing literature, i.e. unaddressed issues and missing recommendations requiring future research. Globally, our aim is to help researchers, patients, research institutions and research ethics boards, reflecting on and dealing with these issues. This paper also aims to inform policy making, and to improve the practice of patient partnership in healthcare research.

## Methods

To conduct our research, we adopted a scoping review methodology. This methodology has the potential to capture “key concept; gaps in the research and types and sources of evidence to inform practice, policy making and research" (p. 8) [15]. Scoping reviews “can be undertaken as stand-alone projects in their own right, especially where an area is complex or has not been reviewed comprehensively before" (p. 194) [16].

According to Arksey and O’Malley [17], there are four reasons to undertake a scoping review:

1. To examine the extent, range and nature of research activity in a particular area;
2. To determine the value of undertaking a full systematic review;
3. To summarise and disseminate research findings; and
4. To identify research gaps in the existing literature.

Reasons 1, 3 and 4 corresponded to our research objectives.

Our review was conducted following the scoping review framework proposed by Arksey and O’Malley [17]. This framework includes 6 stages: 1) Identifying the research question; 2) Identifying relevant studies; 3) Selecting studies; 4) Charting the data; 5) Collating, summarising, and reporting the results; and 6) Consulting with relevant stakeholders. The six stages are reported hereafter.

### Stage 1: identifying the research question

As mentioned, the main objectives of our research project are to map the literature related to the ethical issues associated with patient partnership in healthcare research, and to identify the proposed recommendations to deal with these issues and challenges. Thus, the research question that guided our bibliographic search strategy was “What are the ethical issues associated with patient partnership in healthcare research discussed in the published literature?”

### Stage 2: identifying relevant studies

Two reviewers (AM and JTM) conducted the search and review between December 2017 and December 2018. In order to identify the studies relevant to our review, we interrogated three electronic databases: (1) PubMed (medical, biomedical and life sciences literature); (2) CINAHL (nursing, allied health professions, biomedicine and healthcare literature); (3) EMBASE (biomedical and pharmacological literature). The research queries were formulated according to the specific syntax for each database. We interrogated the title and abstract fields using keywords related to four concepts: “patient”, “engagement”, “research”, and “ethics”.

In our preliminary analysis of the literature, we found out that in some cases the term “community partnership” and “patient partnership” were used interchangeably, and that the term “community” was sometimes used to designate stakeholders that are not usually part of traditional health research teams, including patients, caregivers or user committee members. We thus decided to included “community” as a search term, along with “patient”, but made sure to only include in our study selection the studies addressing patient partnership in research (see Stage 3 of our methodology).

The search terms used are summarized in Table 1. Research results were saved using the bibliographic management software EndNote.

**Table 1:** Search terms

Patient	Engagement	Research	Ethics
Patient* **OR** Communit*	Engage* **OR** Partner* **OR** Collaborat* **OR** Commitment* **OR** Particip* **OR** Involv* **OR** Represent* **OR** Implicat* **OR** Adher* **OR** Centered **OR** Guided **OR** Oriented **OR** Co*author* **OR** Co*investigat*	Research*	Ethic* **OR** Moral* **OR** Integrit* **OR** value* OR model*

### Stage 3: selecting studies

Our research included peer reviewed studies published in English from 2007 to 2017. We initially conducted the search in English and in French (French is the first language of the three authors of the paper), however, we did not identify any article matching our inclusion criteria in the French literature, so we focused only on the English results. We focused on this 10-year window, since our goal was to identify current ethical issues related to the emergent practice of patients being engaged as partners in health research initiatives. Patient engagement in research is indeed a recent approach in healthcare research, which has become more prominent over the past 10 years, notably since research funding agencies around the world have started to support this approach.^1^

AM analyzed the title and abstract of each identified article (*n* = 187) to determine if it addressed the ethical issues associated with patient partnership in research. After removing duplicates (*n* = 45), errata (*n* = 1), commentaries (*n* = 10), clinical studies (*n* = 52), editorial (n = 4), thesis (*n* = 1), poster abstracts (n = 4), erroneously identified references (n = 1), book chapter (n = 1), as well as one article with no accessible full text (n = 1), AM analyzed the full text of all remaining studies (*n* = 67) to exclude those that did not address partnership with patients in research (*n* = 38), such as in the case of patients engaged as research participants, or in the case of patients only engaged as advisors, which does not qualify as patient partnership, according to Carman et al. [1] continuum of patient engagement in healthcare and the patient-as-partner view presented in the background section [2, 5, 6]. We also added references identified from hand search (*n* = 2). At the end of our search strategy, 31 articles remained, which consisted in our final study sample (final *n* = 31). A flow chart of the search results is presented as Fig. 2 in the “Results” section.

### Stage 4: charting the data

After having identified all relevant articles to our review, we collated bibliometric data to qualify our study sample, as suggested by Peters et al. [18]. This data is presented in Table 2, in the “Results” section.

**Table 2:** Description of included studies

	Included studies (***n*** = 35)	
	n	(%)
**Study type**		
Research article	19	54%
Review	9	26%
Theoretical	1	3%
Practical application	2	6%
**Research methodology**		
Qualitative	15	43%
Quantitative	0	0%
Mixed	5	14%
**Country**^**a**^		
USA	17	49%
Canada	5	14%
UK	5	14%
France	1	3%
Switzerland	1	3%
Netherlands	2	6%
**Authorship**		
Patient as co-author	2	6%
Researcher(s) only as authors	29	83%

^a^According to first author affiliation

We proceeded with the full text analysis of each article in our study sample. AM conducted the initial review, and JTM randomly selected 15 references for a second independent review in order to verify intercoder agreement. There was substantial agreement between both reviewers on the identification of the issues in the reviewed articles, and the few disagreements were resolved during one research meeting between AM and JTM.

All issues, barriers and challenges associated with partnering with patients in every steps of the research process were extracted from the selected articles, as well as their associated recommendations for practice, when addressed in the sampled articles. These first-order issues and recommendations correspond to the initial set of codes in our coding process. In line with our scoping review approach, we decided at this point to include all issues, difficulties, barriers and challenges mentioned in our sampled articles, using a broad understanding of what can be considered as an ethical issue. For example, we encountered issues that were deemed at first more practical, such as the absence of compensation offered to patient-partners, but we could also see the underlying ethical aspect to these issues, such as a search for justice and equity between research team members in the case of compensating patient-partners. We thus decided on including all pertinent issues and challenges identified in our sampled articles.

**Fig. 1:**
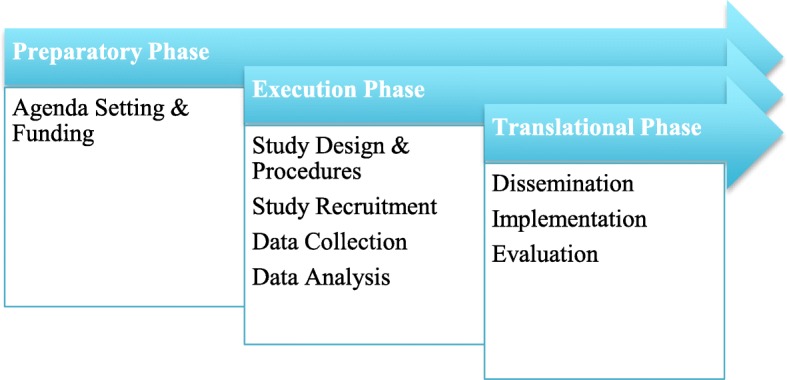
Framework of phases and stages of patient engagement in research, adapted from Shippee et al. 2015, p. 1157

To organize our results, we used Shippee et al. [6] framework of phases and stages of patient engagement in research presented in Fig. 1. As patient partnership aims to engage with patients in all steps of the research process, we figured issues and recommendations about this practice should also be addressed for every research step.

### Stage 5: collating, summarising, and reporting the results

During our review, it was first found that several issues were transversal to all research phases and stages of research and were identified as such by the authors of our reviewed papers. We thus decided to create a separate result section for those transversal issues (see Table 6 in the Results section).

To synthetize our results in a conceptually pertinent way, we regrouped the first-order issues (our initial codes) in second-order themes of ethical issues (see first column of Tables 3, 4, 5 and 6), through thematic analysis [45]. The predominant themes were elaborated using an inductive and iterative process through multiple meetings between research team members (JTM, AM and AB). During this iterative process, all disagreements between the reviewers concerning the classification of the first-order issues into the second-order themes were resolved. In these meetings, we worked on collating and summarizing all identified first-order issues in these second-order themes, organized in the three phases and eight stages framework of patient engagement in research proposed by Shippee et al. [6]. The labels of the second-order themes of issues were also determined collectively and inductively. Results are discussed and presented in summary tables in the “Results” section.

**Table 3:** Issues associated with partnering with patients in the Preparatory phase of research

Themes	Issues identified in the literature	Recommendations identified in the literature
***Research step: Agenda setting and funding***		
**Lack of resources available to support partnership in the early phase of research**	• Researchers may lack financial resources to support full partnership with patients in the early phase of research, such as when determining the research question, before they obtain funding [19–22].	• New funding mechanisms that would recognise the particularities of patient engagement in the phase of research questions development should be created [19]. • Future studies should consider including a full analysis of the resources required for partnering with patients in their research project [20].
	• Researcher might not have enough time to engage in full patient partnership at this early stage of research [19, 21].	• In order to cope with this lack of time and resources, researchers can turn to social media for crowdsourcing, or to investigate community interest for different avenues of research [23].
**Disagreement on priorities**	• Confrontation may arise between researchers and patients when prioritizing research questions or selecting outcomes [24–26].	• Research partners should recognise the difference of objectives between researchers and patients as an opportunity to converge them into synergistic goals [24]. • Research teams should make sure the research question is relevant to all research partners equally, including researchers and patient [27].
	• Different patients may want different research questions to be answered and different outcomes to be measured [11].	• Researchers might select patient partners who are connected with a greater patient community (e.g., through advocacy organizations) and who are able to discuss broad concerns of interest for diverse patients, not just for themselves or their special interests [11].
**Tokenism**	• Challenges for researchers arise when they are faced with a predetermined research topic by funding organizations, while at the same time they are required to implicate patients in the process of research question prioritization. This situation may lead to patient engagement tokenism and inhibit effective implication of patient in the earlier step of research [26]. • Engaging patient in the early stage of the research question development is still uncommon. The participation of patient in this stage of research is commonly limited to consultation rather than full partnership [19]. • Researchers believe that patients aren’t knowledgeable enough to be able to choose research priorities [28]. • Researcher believe that patients lack the expertise to be able to gain global understanding of the whole study [21].	
	• Partners may face long periods of inactivity due to the time required for review after protocol is submitted. Partners who are not appropriately informed about the delays may lose motivation and experience frustration [22].	• Researchers could engage with patients only after funding is secured [22].
**Patients’ conflict of interest**	• During the step of topic solicitation and research question development, challenges may occur when patients focus on their own current health issues and interest while discussing research priorities with researchers [26]. • Patients can anticipate personal benefits as incentive for participating in research, or research ethics committee, which might make them overlook risks for other participants and themselves in the ethical evaluation of the project, in hope of these benefits [21].	• Researchers can encourage patient-research-partners to adopt a broad perspective when expressing their opinion [11, 19, 24, 27].

**Table 4:** Issues associated with partnering with patients in the Execution phase of research

Themes	Issues identified in literature	Recommendations identified in the literature
***Research step: Study design & procedures***		
**Patients exclusion from research stage**	• Due to the lack of patients’ knowledge about research methodologies, their implication in the study design phase may rise concerns about the integrity of scientific methods [28].	• The study design should express a balance between the scientific rigours of researchers and the patient’s suggestions based on their experiential knowledge [27].
	• Some researchers believe that patient partners should not assess the methodological aspects of the study. They believe that the scientific approach is incompatible with the patient approach [28].	• Teams should provide significant training requirement that prepare patients to become research partners [28]. • An effective partnership with patient in the study design phase require that all research materials can be understandable by patient [28].
***Research step: Study recruitment***		
**Challenges in participants recruitment**	• Patient partners may be motivated by the desire to provide benefits to community members, thus they may target as participants individuals whom they think are in the need or deserving services [25]. • When community leaders are partners in research, other community members may feel pressured to enroll as participants in the research [29].	• It is the role of researchers to ensure that all participants are voluntary recruited to participate in research, and that they are not being made vulnerable because of their refusal to participate [29].
***Research step: Data collection***		
**Potential breach of confidentiality**	• Patient partners engagement in research might compromise data confidentiality [25, 30].	• Research teams or research institution should provide ethical research education to patient partners [25].
***Research step: Data analysis***		
**Potential breach of confidentiality**	• Research process could expose patient partners to incidental findings on other patients’ health [25, 31].	

**Table 5:** Issues associated with partnering with patients in the Translational phase of research

Themes	Issues identified in the literature	Recommendations identified in the literature
***Research step: Dissemination***		
**Patients exclusion from research stage**	• Patients are usually excluded from research results dissemination/communication activities (e.g., conference presentations, scientific articles) [13, 32]. • The budget and the time required for the preparation of manuscripts and the dissemination of results are usually under estimated, which could have an impact on the effective implication of patient in this stage of research [33].	• Some researcher encourage patients to disseminate results through social media and patient advocacy groups [13]. • Research teams might also consider alternative dissemination strategies, such as community-forums presentations, non-traditional or locally based media outlets, connecting with local health consortia or community networks [24].
**Disagreement on priorities**	• Conflict may arise between researchers and patient partners concerning the focus of research result dissemination activities and tools. o Researchers usually prefer highlighting generalizable outcomes, which increase the chance of publication and of obtaining future funding, while patient partners usually prefer highlighting results that lead to improved services and/or new or updated public policies [25].	
**Patients’ co-authorship negatively impacting dissemination**	• Medical journals might have a reluctant attitude towards publishing research articles in which patients are identified as coauthors [13].	• Bibliographic databases should add indexing terms that identify research made in partnership with patients, which would facilitate their future use and citation [5].
***Research step: Implementation***		
	n/a	
***Research step: Evaluation***		
**Lack of evaluation framework**	• There is a lack of evaluation framework that could help demonstrating the value and impact of patient engagement in research [13, 28, 33]. • Concerns were raised over the lack of evidence-based policy in support of engaging patients in research, particularly in light of the considerable time, cost and efforts required to embrace ‘effective’ partnership with patients in healthcare research [34].	

**Table 6:** Transversal issues associated with partnering with patients in healthcare research

Themes	Issues identified in the literature	Recommendations identified in the literature
**Challenges in the selection of patient-partners**	• Patient partners may suffer from unstable health conditions, which can jeopardize their engagement in the research project [21, 33, 35].	• Researchers should select patient research partners in stable health conditions [21, 33, 35].
	• Patient partners in research may lose their “lay experience” after multiple previous patient engagement experiences [7]. o This issue is also referred to “pseudo-professionalization” of patients [36].	• Researchers can recruit a mix of new and more experienced patient partners [36].
	• Patient partners experience may not be representative of the larger targeted patients’ population [28]. o When patient partner is a community leader, he may not be perceived as representing his community, lacking legitimacy [29].	
	• Patient partners selection may be biased. o Patient partners are commonly chosen based on their capacity to be articulate, to understand research and to collaborate with researchers and patients, rather than according to the authenticity of their health experience [19, 28, 34]. o Discrimination may occur to the detriment of patients from racial and ethnic minorities [24]. o Commonly, individuals with higher motivation and higher educational level, middle-aged, middleclass, white, are engaged [13, 19, 36]. o Recruitment methods, such as the use of internet and the selection of patient during annual meetings, may cause bias [33].	• Recruiters of patient partners should commit more to diversity, which mean involving different people who face barriers to participation in research [34]. • Funding agencies may formally encourage the inclusion of these underrepresented groups by making it a criterion for funding [24]. o However, as pressures from funders to conduct partnered research with vulnerable communities may help to enhance the representation of these communities, it can also lead to several forms of bias and research protocol violation such as tokenism, pressure on individuals to participate and data falsification [25]. • Random sampling method is less common, although it is the least biased methods since the small number of patients to engage [5].
	• Patients may feel pressured to engage in research. o Pressure can come from being recruited by their own physician [25]. o Peer pressure can come from being recruited by other patients [25] or by community leaders [29].	• Recruitment by peer recruiters can be a solution [25], but also cause for peer pressure, as indicated left.
	• There might be disparity in patients’ access to research partnership. o Some patients consider that the main barrier to their participation in research as partners is the difficulties to learn about available research opportunities [37].	• Patient organisation group may play a role in assisting researchers to reach patients who are difficult to identify and reach [33].
**Absence of a shared vision of patient partnership**	• Research team members may diverge in their comprehension of their respective roles, and of the role of patients, in the research process [22].	• There should be transparency and clarity on the purpose of the study and the process of research [34]. • Research teams must ensure informed consent and provide ongoing clarification of study participation and goals [24]. • Training content should focus on the role of each stakeholder but also should situate these roles in the overall project team [25].
	• Researchers may mistrust the value of patients’ co;ntribution [28]. o Researchers consider patient engagement just as complement or support to the research process and that researchers are ethically the first responsible of the methodological integrity of the research [26]. o Researchers may consider patient engagement as a threat to research integrity [26]. o Researchers can fear losing control on the decisions regarding the research project if patients have a say in it [26].	• Efforts should be made to convince parties of the value of patients’ contribution. o With a concrete curriculum and practical training, patients are capable of contributing to discussions, collaborating to find solutions and impacting the health care system [7].
	• Researchers may challenge patients’ objectivity. o Patient-partners have a tendency to tell their personal story instead of bringing a reflexive opinion [34]. o Patients are considered too emotionally involved [7].	
	• Patient-partners may have misconception of the benefits of their engagement in research [21]. o Patients can expect therapeutic benefits [19, 24] or services [25], from their participation in research activities.	
	• Patient-partners may abandon the research project. o Patient can drop out of research, due to the lack of full disclosure of all expectations related to research [13], or to the varying and fluctuating levels of commitment or the slow translation of knowledge into practice [38].	• There should be transparency and clarity on the process of research [34].
	• Conflict between patient-partners and researchers may arise. o Building an equitable relationship between researcher and patient may be challenging since they have different perspectives [9].	• Conflicts should not be considered as negative. Instead they should be seen as opportunities to learn new way of thinking. Conflicts should be addressed in a manner that recognizes differences in values and skills between both sides [9, 34]. • Researchers do not need to agree with patient perspective. They need rather to remain open to debate and discuss [25, 34].
**Logistical and practical barriers to research partnership**	• Time required from patient partners in research can be a barrier to their continued participation in the project [28]. o Patient bear the burden of frequent and regular meetings [19], which means they have to time and resources for attending [33]. This could create patient frustration toward the length and delays of the research process and the efforts required for attendance [38], and for training activities [5, 32].	• Full disclosure of time required for research partnership is necessary [35]. • Research teams should schedule meetings in accessible locations at suitable times to enable partners to attend [22]. • Research teams should adjust meeting schedule and provide adequate breaks for rest during meetings and research activities in order to accommodate patient health condition [39]. • Research teams can shift their research meeting location to go meet patient partners, which may help to eliminate logistical and practical barriers to patients’ research engagement [24]. • Teams can coordinate research activities with patients’ usual treatment visits (time and location) [35]. o However, the coordination of research activities and usual clinic visit “may lead to conflation of the obligations, risks and benefits of research participation with other clinical or social services they receive” [24]. • When travelling is involved, teams should provide patient partners adequate travel time, as well as appropriate, and comfortable accommodation [39].
	• Individual costs engendered by patient partners for their participation in research activities can be a barrier to their continued participation in the project. o E.g.: Transport costs, parking fees, loss of remuneration, absence of compensation, costs associated with attending international conferences, etc. [28, 36].	• When traveling is involved, teams should cover patient’s travel insurance and reimbursement of expenses [22]. • Covering fees of international travel should include accommodation for patients with severe disabilities (e.g.: special travel arrangements such as supplemental oxygen on flights, or personal assistance) [36]. • *See also recommendations for patient partners’ compensation below*
	• Communication issues between research partners. o Research team members’ usual modes of communication may not suit patient partners (eg. Emails in place of face to face meetings, or phone calls) [22]. o Patient partners may be reluctant to contact lead investigator when in need of more information or updates about the project [22]. o Patient partners may perceive a lack of opportunities to exchange opinions [22].	• Privileging face-to-face contact with patient partners may enhance their implication and support them in their role [22]. • Teams should be in regular contact with patient partners, to provide information about the progress of the study, and/or explanations about particular research phases [22].
**Lack of resources available to support meaningful research partnership**	• Patient engagement is likely to increase the cost and length of time to plan and conduct research [5, 19, 33, 38], as well as the care and attention given to the challenges of the process [39]. Complete and meaningful patient engagement is thus negatively affected by the lack of funding and resources to facilitate partnered research [11, 24, 38, 40]. o There is a lack of funding flexibility for partnered research [32]. o Appropriate training of patient partners and researchers is not recognized by funding agencies and thus not appropriately budgeted [34]. o Pre-engagement activities are required to prepare patients and build trust and relationship, but are usually not covered in funding budgets [32]. o Tight deadlines of funding organizations create pressure on researchers who, under pressure of deadline, may not schedule enough time for training activities for patients. This can inhibit the effective and meaningful contribution of patients in research [26].	• There is a need for more time and more flexible timelines in order to build patient-researcher relationship, trust, and mutual learning processes, as well as to maintain this relation over time [9, 22, 24]. • More time and resources should be granted to training activities [32]. • It behooves to research institution to provide appropriate resource in terms of time, cost and support to offer tailored and relevant training program to research partners [13]. o Research institutions could offer funding for pre-engagement activities leading to patient partnership [26].
**Lack of institutional support offered for partnered research**	• Researchers, especially novices, lack support from their research institution to engage with patients in research [26].	• Research institutions should provide a research infrastructure that fosters partnered research [13], and especially for novice researchers [26]. o For example, research institutions could help in establishing working groups, can provide mentorship opportunities for novice researchers, and access to already developed patients networks and partnerships [26]. • Research institutions should provide long-term support for patient and researchers in order to enhance and maintain effective implication of patients in research [34]. • Research institutions should help teams in the development of evidence-based best practices of patient-engagement in research and may keep a registry of patient engagement activities and initiatives, their results and processes [34].
	• There is a lack of policies facilitating patient engagement in research [33].	• Establish an advisory panel on patient engagement to “ensure the highest patient engagement standards and a culture of patient-centeredness” [41].
	• There is a lack of training on principles and methods of patient engagement research available to both patients and researchers [22, 33]. o When available, training content is most commonly conceived without considering the point of view of patient partners [25]. o Web based training modules may lead to underestimate the value of training by research partners and limit direct discussion [25]. o When available, the length and the complexity of training program can be considered problematic [30].	• Training should be offered to all research partners (researchers and patient) [25]. o There is a need for “training for patients so they understand the research process [and] training for researchers so they understand how to meaningfully engage patients” [28]. o Training should be adjusted to the particularities of the addressed health issue [9, 13, 28, 32, 33]. o Training should be “…easily accessible, both conceptually and logistically to all partners…” [24].
	• Research professionals (other than leading investigators) don’t feel recognized and/or rewarded for the efforts made to involve patient partners in research [22].	
	• Research ethics and research integrity policies and practices are not adapted to the particularities of partnered research. o Traditional models of research integrity, including those typically monitored by institutional review boards, focus on protecting the rights of individual research subjects enrolled in research, but not on protecting patient partners in research [42]. o Ethic guidelines are predetermined without patient partner consultation [25]. o Research ethics education may not take into account the particularities of partnered research and its unique ethical issues [25].	• Training content, especially ethical education, should take into account the input from patient partners since they have more specific knowledge about the vulnerabilities of the targeted populations [25].
**Power differentials between research team members inhibiting or pressurising patients’ contribution**	• Power differentials between research team members, especially between patient partners and researchers, may inhibit patient contribution and/or lead to conflicts [7, 22, 25, 34, 39].	• The success of partnership requires mutual commitment to equitable power sharing and open communication about the requirements of the collaborative process [25]. • Power and ownership of the research process between researchers and patient partners need to be clearly addressed to allow patients to discuss, challenge, or reject the opinions of principal investigators [39]. • Engagement research should be democratized. In order to balance power, researchers need to reduce power behaviors and to learn behaviors that reinforce the capacity of patient partners. Equal and constructive partnerships do not occur immediately but are built by developing trust and self-confidence over a longer period of time [22]. • Relational empowerment: Everyone profits from the collaboration [22].
	• A pre-existing clinical relationship between researcher and selected patient partner may create a potential conflict of interest in the conduct of the research project [25] and inhibit patient partners contribution.	• *See recommendations in the patient selection section,* Table 2
	• Patient partners in research may feel overburden by their responsibilities in the research process. o When patients or community organizations collaborate with researchers, they “may feel overburdened by academic partners’ requests for input on formal products such as grant proposals or manuscripts, particularly if they have a limited number of paid staff members” [25].	
**Tokenism**	• Patient engagement may be tokenistic [5, 28, 38, 43].	• The relation with patient must be genuine and the contribution of patient should be effectively considered [34]. • Researchers should give patient partners the opportunity “to have say and to be empowered in their contribution to the research process” [13, 34]. • Patient/public collaborators should be treated as valued members of the research teams, and not just as tick boxes. The more they are incorporated into the team, the less likely they are to feel just like additional study subjects [44].
	• Patient partners engagement and contributions are limited to specific research activities [19, 28, 36].	• Research teams should develop a checklist that identifies roles or tasks in order to ensure the implication of patients and track their activities [22].
**Patients and/or researchers insufficient knowledge weakening research partnership**	• The lack of scientific and methodological background of patient partners is problematic for researchers [21, 28].	• Pairing new patient partners with experienced patient partners (*buddy system)* could help in the knowledge transfer and the assistance of new patient partners [34, 36].
		• While patient engagement in research should be encouraged, principal investigators should maintain authority in developing protocols to ensure scientific rigor [11].
	• Researchers may lack knowledge on partnered research principles and process needed to be able to engage with patients in a meaningful way [38].	• *See recommendations in “lack of institutional support”,* Table 6
**Conflict of interest affecting research team members**	• Conflicting interests of researchers may influence their contribution to research. o Researchers often bears dual roles as researchers and healthcare provider for patient(s) engaged in research. This can cause competing responsibilities and conflation of obligations [24, 36].	• Potential conflicts of interests must be discussed in advance with parties [11]. • Researcher must consider the separation between their role as treating doctor in the clinic and as a researcher in the context of research project [36]. • Research team could design a consultant doctor for patient partners if they have any health concerns during the research project [36].
	• Conflicting interests of patient partners may influence their contribution to research. o When patient partner is also a healthcare provider or a service provider, they may be in a situation of conflict of interest due to the dual role they play. In this case, “their nonresearch roles may value social and ethical norms that differ from research norms, creating ethical ambiguity or conflicts of interest” [25]. o Patient partners or patient organizations may be in relation with industry groups, which can result in conflict of interest [33]. o Patient partner can also be patient advocate, or member of a patient advocacy organization, which can result in conflict of interest [33].	
**Harm caused to patient-partners**	• Patient-partners engagement in research may cause them harm. o Their participation might cause a resurfacing of bad memories [44]. o Discussions on sensitive topics may pose challenges, with the potential for harm to patient collaborators [44].	• Meaningfully implication of patient in the research team may help to reduce bad memories resurfacing [44]. • Teams may provide a psychotherapist for patient, for assistance if needed during their partnership [39]. • “special attention is required for adequate rest, nutrition, debriefing, and emotional support throughout the research process” [39]. • Patients, as opposed to patient representatives or lay members, should be engaged in research ethics committees to ensure that patients’ voices are heard [21]. o Ethics committee could derive ethics guidelines from patient’s preferences: Through interactive discussion session they can gather their perspective on research ethics issues [37]. • Researchers require specific ethics education relevant to patient engagement [25].
**Absence and/or impact of patients’ compensation**	• Patient-partners may not be compensated for their engagement in research projects [39]. o This could be the result of absence of appropriate funding for partnered research [28]	• Research teams/institutions should provide appropriate honoraria to the critical expert knowledge that patient/survivor consultants or partners provide to research [39]. o “the honorarium was equivalent to a physician or researcher fee for participation in a focus group” [39]. • Research teams/institutions often reimburse food or other costs to motivate participation and commitment to research [35].
	• Patient-partners could be motivated by financial reward expectation for their implication in research [13].	• Research teams might not disclose in advance they offer patient’s financial compensation in order to mitigate undue influence for participation [39].
	• Concerns arise about the impact of the remuneration on the individual situation of patient-partners, such as its potential effects on the government benefits they receive [13].	
**Traditional research culture as a barrier to partnered research**	• Traditional research culture is a barrier to researchers and patient’s engagement in partnered research. o Researchers are trained and used to write grants and set research priorities by themselves. Implicating patients can be challenging for them [19, 26, 28]. o It is difficult to build a research partnership with patients from countries or culture where “participatory research is less recognized and physician–patient relationships are traditionally more paternalistic” [36].	• There is a need for a shift in the research culture and a necessity to rethink traditional research methods in order to make them more opened and favorable to partnered research [28].

We also used a data extraction chart to visually map the literature, identify recurring issues and recommendations, as well as to help us identify gaps and unaddressed challenges and issues. In the chart, we indicated the presence of all themes of issues in all sampled articles, regrouped in stages of research. Note that the presence of a theme of issue, as indicated by a “x” in the chart, does not qualify the information about this issue, but only the presence of the issue in the article reviewed, might it be discussed in depth, or only in passage. The extraction chart is provided in Additional file 1.

### Stage 6: consultation exercise

Although the consultation stage is optional in Arksey and O’Malley’s [17] framework, we did integrate a consultation exercise with a committee composed of stakeholders and practitioners with expertise on patient partnership in research. According to Arksey & O’Malley [17], the consultation exercise is an “additional, parallel element” of the scoping study framework that can “inform and validate findings from the main scoping review” (p. 9). Since the overarching aim of this research was to inform policy making about the ethical issues associated with the practice of patient partnership in research and to make recommendations to researchers, patients, research institutions, etc. to improve its practice, we assumed that working with stakeholders would increase the relevancy of results, help identify unaddressed ethical issues, and facilitate knowledge transfer.

Our stakeholder committee was composed of two researchers experienced in conducting research with patients, two patient-partners in research, two representatives from distinct research ethics boards from healthcare research institutions in Quebec, Canada, an ethicist from the Fonds de recherche du Québec (FRQ), the main public funding research agency in the province, and an ethics expert from the Canadian Institutes of Health Research (CIHR).

A first meeting was organized prior to the reviewing process and aimed at presenting the objectives of the research and to have a better idea of the needs of each stakeholder in the matter of knowledge transfer following our study. A second consultation was organized after the literature review was completed. For that, we sent all members of the stakeholders committee the results of our review by email, and asked them to comment it and identify gaps, i.e. unaddressed issues and challenges, based on their own experience. AM and JTM collected and then proceeded to code all first-order issues, recommendations and comments from the written answers received. We then collated the results in a table, eliminating redundancies and unoriginal issues, i.e. those that were already covered in the scoping review. We synthetized all the new first-order issues and recommendations conveyed by the stakeholder committee, as well as the comments that we found pertinent and enlightening, i.e. those giving a different perspective or adding an important precision to the issues already identified in the review. The results of this consultation are presented in Table 7, in the “Results” section.

**Table 7:** Results from the stakeholders’ consultation exercise

Phase of research	First-order issues and/or recommendations already identified in the literature	New first-order issues, recommendations or comments from the stakeholder committee
**Preparatory phase**	n/a	**New recommendation**: Major funding agencies should provide training and information on patient partnership in research.
	n/a	**New issue**: Lack of engagement due to prior negative experiences. Researcher may have had, or heard of, negative prior experiences and thus be reluctant to engage patients in the preparatory phase of his research project.
	n/a	**New issue**: Overemphasis of certain research themes due to patients being engaged in research. Common conditions such as cancer or diabetes may be overemphasized in research, because of the prevalence and availability of patient partners.
	n/a	**New issue**: Overemphasis of certain types of research. Involving patient partners might reduce the place of fundamental research in research programs because of an emphasis on obtaining results that can rapidly be transferred to care.
**Execution phase**	n/a	**New issue**: Efficiency. Researchers may worry that implicating patient partners may alter the dynamics and thus the efficiency of research meetings.
	n/a	**Comment**: An important avenue for research is on patients’ responsibility in the matter of research ethics and research integrity.
**Translational phase**	Issue: Patients are usually excluded from research results dissemination and communication activities.	**New recommendation**: Committee recommended to anticipate this issue by discussing the planned dissemination activity and the patient involvement in them early on.
	n/a	**Comment**: Committee pointed out it would be useful to do more research on how patients see and prioritize research dissemination activities. They may help to improve research knowledge transfer to population.
**Transversal issues**	Issue: Patient partners may suffer from unstable health conditions, which can jeopardize their engagement in the research project	**Comment**: Committee pointed out that it may not always be possible to anticipate risk of patients relapsing or getting another health issue in the future. Inclusion criteria may be too narrow and discriminatory if one of the selection criteria is to only include patients in stable health conditions. Mitigation strategies or reasonable accommodations should instead be put in place if patients suffer from unstable conditions during the research project.
	Recommendation: Selection of patient partners in stable health condition.	
	Issue: Patients may lose their “lay experience” after multiple previous patient engagement experiences.	**New recommendation**: Committee suggested that when patients represent a community, they can be encouraged to consult with their community regularly.
	Issue: Representativity. Patients may not be representative of the larger patient’s population.	**New recommendation:** It was suggested that patients should indicate whether they speak for themselves or they represent a community when engaged in a research project.
	Issue: Patient partners may have misconception of the benefits of their engagement in research (they might expect therapeutic benefits, or services).	**New recommendation**: Committee recommended research team discuss this issue earlier on in order to clear all misconceptions patients may have.
	Issue: Communication issues between research partners	**New comment**: Committee felt that these communication issues are sometimes caused by the use of technical language or medical or health system jargon by researchers.
	Issue: Conflict between patients and researchers may arise	**Comment**: Committee indicated that conflict may also happen when a patient partner is particularly vocal or particularly vindictive or demanding.

## Results

**Fig. 2:**
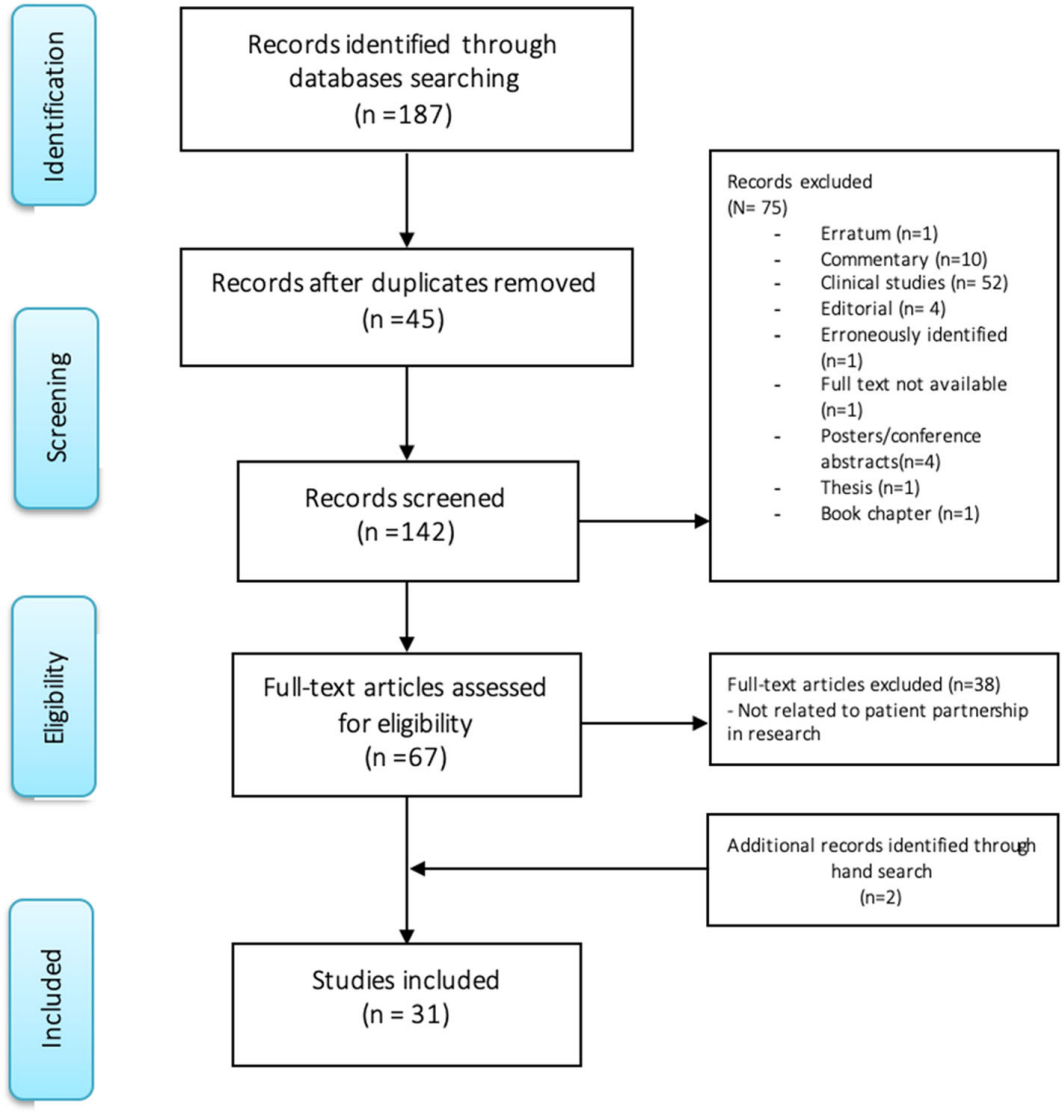
Flow chart of search strategy and search results

A flow chart of the search strategy and search results is presented in Fig. 2. Following, we present a description of our sampled articles, in Table 2.

Interestingly, only few papers in our sampled articles reflected the patient’s perspective on the ethical issues associated with patient partnership in research (for example De Wit et al., 2013), which means that the following results are thus principally the view of researchers on the topic.

We hereafter present the results of our review of the ethical issues and challenges associated with patient partnership in research identified in our sample articles, as well as associated recommendations, for all steps of the research process. We also added a table presenting issues that are transversal to all phases and stages of research. Findings are presented in complete tables, in which first-order ethical issues (identified with a black dot) are regrouped in second-order themes of ethical issues, following our thematic analysis.

**Fig. 3:**
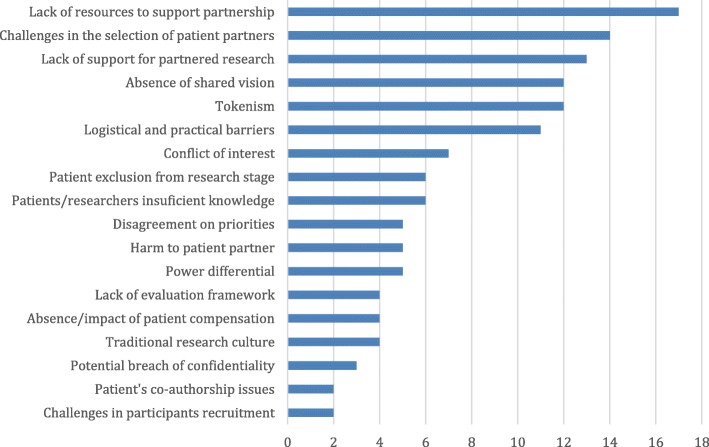
Themes of ethical issues associated with partnering with patients in healthcare research –Occurrences in the literature

In the following Fig. 3, we present a synthesis of the second-order themes of ethical issues identified in our literature review. We have listed all the second-order themes of ethical issues presented in our result tables together, independently of the different phases and steps of research, and regrouped similar themes, to obtain a final list of themes of ethical issues, as well as the importance given to each of them in the reviewed literature, measured in occurrences. More precisely, the theme “lack of resources to support partnership” was associated with the preparatory phase of research (Table 3) but was also mentioned by authors as a transversal issue (Table 6). In Fig. 3, we regrouped both themes, and removed redundancies in order to only count one occurrence per reviewed article. We did the same regrouping for the theme “tokenism” (from Table 3 and Table 6); the theme “conflict of interest” (from Table 3 and Table 6); the theme “patients’ exclusion from research stage” (from Table 4 and Table 5); and the theme “disagreement on priorities” (from Table 3 and Table 5).

### Unaddressed issues and recommendations

Following our consultation with the stakeholders committee, we were able to identify the followings gaps in the literature, i.e. the missing ethical issues and/or the lack of associated recommendations for an ethical partnership with patients in healthcare research. These also include the issues that have been identified and presented in the result tables, but that haven’t been the object of much research or discussion in the literature we scoped. We collated the new first-order issues, new recommendations and the comments obtained in this consultation exercise in the Table 7.

The 4 new issues highlighted by the stakeholder committee were coherent with the 18 second-order themes of issues presented in Fig. 3. Indeed, the issue of the lack of engagement due to prior negative experiences of engagement researchers may have had or heard from is encompassed by the second-order theme “absence of a shared vision of patient partnership”. The two new issues about the influence of patient engagement in the early phase of research on the research agenda and types of research undertaken are coherent with the second-order theme “disagreement on priorities”. Finally, the last new issue about patient-partnership impacting team efficiency is encompassed by the second-order theme “logistical and practical barriers to research partnership”.

## Discussion

We have identified a total of 58 first-order ethical issues and challenges associated with partnering with patients in healthcare research from the main scoping review (see Tables 3, 4, 5 and 6). Some issues were specific to the Preparation phase of research (*n* = 11), the Execution phase of research (*n* = 6) and the Translational phase of research (n = 6). However, the majority of the issues identified in our scoping review were classified as transversal issues (*n* = 35), pertaining to all steps and phases of research or to the general practice of engaging with patients as partners in different healthcare initiatives. We believe most of these transversal issues, such as the absence of compensation for patients, and the lack of training on patient-partnership for team members, to be also common to other spheres of patient-partnership initiatives, such as engaging with patients in governance committees, in quality improvement initiatives, or in healthcare education. Indeed, it appears that a lot of these issues are not specific to partnered research, but rather to the establishment of a sincere, satisfying, and functional partnership between patients, healthcare professionals and administrators.

We regrouped the first-order issues in 18 second-order themes of ethical issues. As presented in Fig. 3, the most prominent second-order themes of issues associated with partnered research found in the literature, i.e. those that presented the most occurrences in our sampled articles, were: a) the lack of resources available to carry research in partnership with patients; b) the challenges in the selection of patient-partners; c) the lack of support offered to research teams and patients; d) the absence of a shared vision on research partnership with patients; e) a tokenistic patient engagement in research; and f) the logistical and practical barriers to partnered research.

The results from the consultation exercise added interesting precisions and new perspectives on the issues and recommendations already scoped in the literature review, and also added 4 new first-order issues to the list (see Table 7). We were particularly interested in the identified unaddressed issues relating to patient engagement in the early phase of research, notably in the research agenda setting. It was brought forward by the stakeholder committee that patient engagement in the beginning of a research project may favor or overemphasis certain themes of research, on more common pathologies such as cancer or diabetes, and could also favor certain types of research, which would be more focused on getting results that can be quickly transferred to care, as opposed to fundamental research. These important issues are definitely to be explored further.

### Ethical frameworks for patient partnership in research

When analyzing the results of our review, it appeared to us that numerous issues and challenges associated with patient partnership in healthcare research related to ethical frameworks that expanded way beyond the traditional research ethics framework that is generally used to reflect, anticipate, prevent and deal with the ethical issues associated with a more traditional research approach. Indeed, the practice of partnering with patient in research, i.e. of doing research with, and not on, the patient involves changes in the way we reflect on the ethical issues associated with this research approach. In a more traditional research approach, patients are usually involved only as research participants. The ethical issues surrounding the relationship between the research and the patients in this case are prevented and dealt with research ethics principles and processes, often managed through the process of obtaining an ethics certificate from a research ethics board. The general goal is to protect research participants from harm. But when we switch to doing research in partnership with patients, the issues are more diverse. Of course research participants are still to be protected under the principles and processes of research ethics, but there are added goals aimed in the ethical reflection on the issues engendered by partnered research, such as to protect the partnering relationship, to assure the best contribution of all actors, as well as to protect patient partners in research. Research ethics principles and processes focused specifically on protecting research participants are thus in most cases inapplicable or unrelated to the diverse and numerous issues specifically related to partnered research we have identified in our scoping literature review. This broadening of the conceptualization of ethics in the context of partnered research appeared to us as an important contribution of our review, and we thus attempted at a different categorization of the first-order ethical issues according to the areas of ethics they related to, and based on the definitions we propose hereafter. This conceptualization was not another step into the thematic analysis, aiming at reducing again the 18 second-order themes into a potential third-order classification. Differently, we wanted to present frameworks that could help researchers, patients and practitioners reflect on the variety of issues associated with the recent practice of patient partnership in research found in the literature, and to show that the traditional framework used in research was in most cases unhelpful or inapplicable. This different categorization was developed inductively and qualitatively, during meetings of the research team (JTM, AM and AB).

**Fig. 4:**
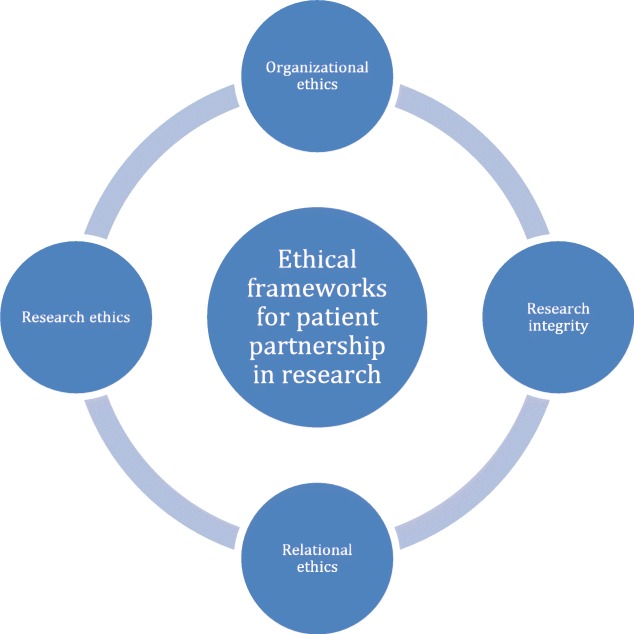
Ethical frameworks for patient partnership in research

We have found that the 58 first-order ethical issues and challenges associated with patient partnership in research identified in our literature review, as well as the 4 issues brought forward by the consultation exercise, can be related to four ethical frameworks that we have identified as: 1) Research ethics; 2) Research integrity; 3) Organizational ethics; and 4) Relational ethics. These are illustrated in Fig. 4.

As mentioned above, research ethics is interested in the ethical issues raised by involving patients as research participants, and focuses on respect for participants, autonomy of patients, concern for welfare, protection of participants from risks, justice and issues associated with vulnerability. Principles of research ethics are included in national and institutional guidelines and are implemented by institutional research ethics boards (REBs) and researchers. In our scoping review, we have identified only 5 issues (*n* = 5) associated with research ethics. For example, some researchers are worried partnering with patient in research might compromise data confidentiality [25, 30], or may expose patient partners to incidental findings about patient participants [25, 31].

Research integrity is concerned with the way scientific research is carried out, and is focused on scientific rigour, honesty, reliability, referencing and plagiarism, working out authorship, conflicts of interest management, grant application and fund management, for example. Research integrity is the responsibility of researchers and research professionals, as well as research institutions, and guided by national and institutional policies and guidelines. In our review, we have identified 15 issues and challenges to partnered research associated with research integrity. For example, some researchers worry that the lack of patients’ knowledge about research methodologies may raise concerns about the integrity of scientific methods [28], or that patients’ potential conflicts of interest may influence their contribution in the research process [25, 33]. Research ethics and research integrity are now often regrouped under the umbrella of the concept of responsible conduct of research (RCR). Added together, they account for roughly a third (*n* = 20) of the issues identified in our review.

Differently, organizational ethics focus on the practices, programs, structures, reward allocation, and leadership needed to encourage the ethical and responsible conduct of employees and managers in organizations, and to foster ethical and responsible relationships with stakeholders. It is interested in ethical decision-making and behavior from organizational actors, and the management of misconducts. It is carried out in organizational mission, values and culture, embodied in codes of ethics and value statements. In the context of patient partnerships in healthcare organizations, it is not always clear how patient partnership initiatives are supported. Some health organization have dedicated structures responsible for supporting patient engagement, but these structures may fall under different organizational functions and hierarchical levels, and often lack resources, legitimacy, or leadership. Half of the issues and challenges identified in our review (*n* = 30) can be associated with a broad view of organizational ethics, most of them being practical, logistical and processual issues. These include, for example, the lack of support offered to researchers and patients to carry out partnered research, the lack of resources available to foster meaningful partnership, the disparity in patients’ access to research partnership, and the question of the compensation of patient-partners.

Relational ethics focuses on the relationships and the context of the connections between individuals, and highlight values and concepts such as mutual respect, engagement, environment and embodiment [46, 47]. This view is mostly developed in nursing literature [48], but we found it to be quite suited and useful to reflect upon the issues relating to the ethics and the quality of the relationship fostered with patient-partners, within research teams and research institutions, and on the quality and the sincerity of the engagement of patients in the research process. We have identified several issues linked with this concern in our review (*n* = 12), mostly relating to an unmeaningful or insincere partnership being established with patients in the context of research, or to the absence of a shared vision of patient-partnership between members of the research team. For example, it has been discussed that some researchers mistrust the value of patients’ contribution [28], or find that patients aren’t knowledgeable enough and lack the expertise to contribute to research [21, 28], and that patient engagement in research can be tokenistic [5, 28, 38, 43]. For these reasons, patients are often excluded from certain phases of research. Although our review was focused on patient engagement in research, for the most part the issues relating to relational ethics might also be present in other spheres of patient engagement, such as in governance and/or teaching activities. Developing a shared vision and meaningful and ethical relationships with patient-partners is the responsibility of researchers, research professionals, and administrators, but patients also influence these relationships and share expectations.

As mentioned before, the issues mentioned in our review fall under the responsibility of different and often unrelated actors. But most importantly, there is often a lack of coordination between the actors and organizational structures responsible for research ethics, research integrity, organizational ethics and fostering qualitative and ethical relationships with patients in the context of patient partnership initiatives. Indeed, these responsibilities fall under different departments, committees, managers and professionals. For example, organizational ethics is not much developed in Quebec healthcare organizations. Issues linked to organizational ethics are often carried through by clinical ethicists who are sometimes unaware of patient engagement initiatives, especially the ones taking place in the context of research, and not coordinated with research ethics board, or patient engagement support staff. Unfortunately, this lack of coordination creates silo effects, and can exacerbate the ethical challenges research professionals and patient-partners may encounter. The lack of a more coordinated, systemic and strategic management of patient engagement initiatives, including partnered research, in healthcare organizations can prevent the ethical development of this recent research approach. In this context, we anticipate that the traditional culture of research, conducted without engaging patients, may find back its old rights. If policy makers, administrators, health professionals and patients believe patient partnership in research is an approach worth pursuing, it appears that changes will have to happen in its management by health organizations and institutions in order to ensure patient engagement and partnership initiatives in research are carried through in the most ethical way.

### Limitations and future research

This review was limited by a small number of studies that specifically addressed the ethical issues and challenges of partnering with patients in healthcare research. Given the growing interest in this approach and the institutional pressures to engage with patients in research, we believe more empirical research will be needed on the ethical issues raised by this research approach.

Some aspects of the search strategy chosen to conduct our study might also have brought some limitations. First, we chose to focus on the higher level of patient engagement in research brought forward in the literature and in practice, which is the level of patient partnership in research. This focus led us to exclude articles that did address the topic of patient engagement in research, but not to the level of patients being engaged as partners in research. We excluded 38 articles on that criteria, such as papers focusing on patients being engaged as advisors in research or being consulted in research projects. It is possible that some other potentially interesting ethical issues associated with the general practice of patient engagement in research may have been found in these excluded papers. Also, the focus on the 2007–2017 window may potentially have excluded some early papers addressing the ethical issues of patient partnership in research, published before that timeline. However, our preliminary search lead us to specifically focus on that timeline, since we hadn’t found papers corresponding to the search criterion before that. Also, we chose in our review to focus on peer-reviewed articles, but this choice came with some limitations, as it made us exclude other types of articles, such as commentaries, which often add insights into ethical issues associated with a research project. Moreover, our results might be specific to certain national context, namely partnered research in the US, Canada and the UK, since 77% of our sampled articles were authored by scholars from these countries. Also, our review reflected both patients and researchers view on patient partnership initiatives in research in the available literature, however, only few papers from our sampled articles highlighted the view of the patients’ side of the partnership, as mentioned in the Results section. We thus believe there is a need for more research to better grasp the opinions and views of patient-partners on the ethical issues and challenges of partnered research.

We also wish to acknowledge a bias towards the positive value of patient engagement in research. Both JTM and AB are currently engaged in research initiatives conducted in partnership with patients, and are convinced of the importance of this approach, although sometimes critical of the way it is carried through in practice.

Finally, one of the contributions of this scoping review was to offer a conceptual framework of the categories of ethical issues that could inform future empirical research. The proposed framework, as well as other findings from this review, such as the gaps identified in the literature, should be further explored to ensure a more comprehensive and in depth understanding of the ethical issues related to patient engagement in healthcare research. This could be done through interviews and/or surveys methods, conducted with researchers and patient-partners themselves, for example. Further exploration should lead to more specific recommendations for policy making.

## Conclusion

We have identified 58 first-order ethical issues and challenges associated with the patient partnership in research approach in our scoping review of the literature. These were regrouped in 18 second-order themes of ethical issues. In our consultation exercise with a committee of stakeholders, we have identified another 4 first-order issues that complemented the findings from our main scoping review. The results of our review show that most issues are transversal to all phases and stages of research, and that many of these issues could also apply to the practice of patient engagement or patient partnership in other spheres of healthcare such as governance, quality improvement, and education. Our results also show that the ethical issues associated with partnered research are more diverse than the issues usually associated with a more traditional approach to research, conducted without engaging patients. The usual discussion of how we address ethical issues in research is based on the assumption that patients, as research participants, must be protected from harm. However, doing research with, and not on, patients involves changes in the way we reflect on the ethical issues associated with this research approach. We thus propose to broaden the ethical discussion on partnered research to not only rely on a research ethics framework, but to also frame it within the frameworks of research integrity, organizational ethics and relational ethics.

## Supplementary information

**Additional file 1.** Ethical issues identified in individual studies.

## Data Availability

All data generated or analyzed during this study are included in this published article.

## Footnotes

1 For example, in the US, the PCORI Institute was founded in 2010, and the National Center for Advancing Translational Science from the NIH was founded in 2012, and they both supports since 2012 research initiatives on patient and community engagement. The Strategy for Patient-Oriented Research (SPOR) from the Canadian Institutes on Health Research was created in 2011, and since then it supports, and funds partnered healthcare research in Canada.
