# Supplementary material for: Partnering with patients in healthcare research: a scoping review of ethical issues, challenges, and recommendations for practice
Source: BMC Med Ethics. 2020 May 11;21:34. doi: 10.1186/s12910-020-0460-0 (PMC7216517; doi:10.1186/s12910-020-0460-0)
Supplement: Supplementary file 1 — Additional file 1. Ethical issues identified in individual studies. [file 12910_2020_460_MOESM1_ESM.docx]

**APPENDIX:** Ethical issues identified in individual studies

| References | Research stakeholders | | | | | | Themes of ethical issues associated with partnering with patients in healthcare research | | | | | | | | | | | | | | | | | | | | | | | |
| --- | --- | --- | --- | --- | --- | --- | --- | --- | --- | --- | --- | --- | --- | --- | --- | --- | --- | --- | --- | --- | --- | --- | --- | --- | --- | --- | --- | --- | --- | --- |
|  |  |  |  |  |  |  | Transversal ethical issues - Partnering with patients in research | | | | | | | | | | | | Ethical issues associated with specific research phase | | | | | | | | | | | |
|  |  |  |  |  |  |  |  |  |  |  |  |  |  |  |  |  |  |  | Preparatory phase | | | | Execution phase | | | | Translational Phase | | | |
|  |  |  |  |  |  |  |  |  |  |  |  |  |  |  |  |  |  |  | Agenda setting & funding | | | | Study design & procedures | Study recruitment | Data collection | Data analysis | Dissemination | | | Evaluation |
|  | Patient | Researcher | Research institution | Funding organism | Research filed organisation | Medical journal editors | Lack of resources | Lack of support | Tokenism | Traditional research culture | Conflict of interest | Compensation | Logistical and practical barriers | Absence of shared vision | Power differential | Patients/researchers lack knowledge | Harm to patient partner | Selection of patient partners | Lack of resources | Conflict of interest | Tokenism | Disagreement on research priorities | Reluctance to implicate patients | Challenges in participants recruitment | Potential breach of confidentiality | Potential breach of confidentiality | Exclusion of patients | Disagreement on priorities | Patients’co-authorship issue | Lack of evaluation framework |
| Allen et al. (2017) | x | x |  | x |  |  | x | x |  |  |  |  |  | x |  |  |  |  |  |  |  |  |  |  |  |  |  |  |  |  |
| Amirav et al. (2017) | x | x |  |  |  |  | x |  | x |  |  |  | x | x |  | x |  |  |  |  |  |  |  |  |  |  |  |  |  |  |
| Anderson et al. (2012) | x | x | x |  |  |  |  | x |  |  | x |  |  | x | x |  | x | x |  |  |  | x |  | x | x | x |  | x |  |  |
| Aungst et al. (2017) | x | x |  |  |  |  |  |  |  |  |  |  |  |  |  |  |  |  | x |  |  |  |  |  |  |  |  |  |  |  |
| Carroll et al. (2017) | x | x |  |  | x |  |  | x | x | x |  | x | x | x |  | x |  | x |  |  | x |  | x |  |  |  |  |  |  | x |
| Chiu et al. (2013) | x | x |  |  |  |  | x |  |  |  |  | x | x |  | x |  | x |  |  |  |  |  |  |  |  |  |  |  |  |  |
| Concannon et al. (2014) | x | x |  |  |  |  | x | x |  |  |  |  | x |  |  |  |  |  |  |  |  |  |  |  |  |  | x |  |  |  |
| de Wit et al. (2017) | x | x |  |  |  |  |  |  | x | x | x |  | x |  |  | x |  | x |  |  |  |  |  |  |  |  |  |  |  |  |
| Demian et al. (2017) | x | x |  |  |  |  | x |  |  |  |  |  |  |  |  |  |  |  |  |  |  |  |  |  |  |  |  |  |  |  |
| Dillon et al. (2017) | x | x |  |  |  |  |  |  |  |  |  |  |  |  |  |  |  |  |  | x |  | x | x |  |  |  |  |  |  |  |
| Domecq et al. (2014) | x | x |  | x |  |  | x |  | x |  |  |  | x |  |  |  |  | x |  |  |  |  |  |  |  |  |  |  | x |  |
| Ellis and Kass (2017) | x | x |  | x |  |  | x |  | x | x |  |  | x | x |  |  |  | x | x |  | x |  |  |  |  |  |  |  |  |  |
| Forsythe et al. (2014) | x | x | x |  |  |  | x | x |  |  | x |  | x |  |  |  |  | x |  |  |  |  |  |  |  |  | x |  |  | x |
| Frank et al. (2015) | x | x |  |  |  |  |  | x |  |  |  |  |  |  |  |  |  |  |  |  |  |  |  |  |  |  |  |  |  |  |
| Haywood et al. (2015) | x | x | x |  |  | x | x | x | x |  |  | x |  | x |  |  |  | x |  |  |  |  |  |  |  |  | x |  | x | x |
| Haywood et al. (2017) | x | x |  | x |  |  | x | x | x |  |  |  |  | x | x | x |  | x |  |  |  |  |  |  |  |  |  |  |  | x |
| Islam et al. (2014) | x | x |  |  |  |  |  |  |  |  |  | x | x |  |  |  |  | x |  |  |  |  |  |  |  |  |  |  |  |  |
| Isler and Corbie-Smith (2012) | x | x | x | x |  |  | x | x |  |  | x |  | x | x |  |  |  | x |  | x |  | x |  |  |  |  | x |  |  |  |
| Khodyakov et al. (2016) | x | x |  |  |  |  |  | x |  |  |  |  |  |  |  |  |  |  |  |  |  |  |  |  |  |  |  |  |  |  |
| Mamzer et al. (2017) | x | x |  |  |  |  |  |  |  |  |  |  |  |  |  |  |  |  |  |  |  |  |  |  |  | x |  |  |  |  |
| Marlettet et al. (2015) | x | x |  |  |  |  |  |  |  |  |  |  |  | x | x |  |  | x |  |  |  |  |  |  |  |  |  |  |  |  |
| Mullins et al. (2012) | x | x |  | x |  |  | x |  |  |  | x |  |  |  |  | x |  |  |  | x |  | x |  |  |  |  |  |  |  |  |
| Pollock et al. (2014) | x | x |  |  |  |  |  |  |  |  |  |  |  |  |  |  |  |  | x |  |  |  |  |  |  |  |  |  |  |  |
| Rakic et al. (2017) | x | x | x |  |  |  |  |  |  |  |  |  |  | x |  | x | x | x | x |  | x |  |  |  |  |  |  |  |  |  |
| Robillard and Feng (2017) | x | x | x |  |  |  |  |  |  |  |  |  |  |  |  |  | x | x |  |  |  |  |  |  |  |  |  |  |  |  |
| Rose (2014) | x | x |  |  |  |  |  |  | x |  |  |  |  |  |  |  |  |  |  |  |  |  |  |  |  |  |  |  |  |  |
| Ross et al. (2010) | x | x |  |  |  |  |  |  |  |  |  |  |  |  |  |  |  | x |  |  |  |  |  | x |  |  |  |  |  |  |
| Siassakos et al. (2015) | x | x |  |  |  |  |  |  | x |  |  |  |  |  |  |  | x |  |  |  |  |  |  |  |  |  |  |  |  |  |
| Sofolahan-Oladeinde et al. (2017) | x | x | x | x |  |  | x | x |  | x |  |  |  | x |  |  |  |  |  | x | x | x |  |  |  |  |  |  |  |  |
| Westfall et al. (2017) | x | x | x |  |  |  |  | x |  |  |  |  |  |  |  |  |  |  |  |  |  |  |  |  | x |  |  |  |  |  |
| de Wit et al. (2013) | x | x | x | x |  |  | x | x | x |  |  |  | x | x | x |  |  |  | x |  | x |  |  |  |  |  |  |  |  |  |
